# Analysis of receptor tyrosine kinase genetics identifies two novel risk loci in GAS6 and PROS1 in Behçet’s disease

**DOI:** 10.1038/srep26662

**Published:** 2016-05-25

**Authors:** Jieying Qin, Lin Li, Donglei Zhang, Hongsong Yu, Handan Tan, Jun Zhang, Bolin Deng, Aize Kijlstra, Peizeng Yang

**Affiliations:** 1 The First Affiliated Hospital of Chongqing Medical University, Chongqing Key Laboratory of Ophthalmology and Chongqing Eye Institute, Chongqing, People’s Republic of China; 2 University Eye Clinic Maastricht, Maastricht, The Netherlands

## Abstract

The TAM kinase (Tyro3, Axl, Mer) and its two ligands (Gas6 and protein S) have been shown to play an important regulatory role in the innate immune response. The present study aimed to investigate whether the tag single-nucleotide polymorphisms (tag SNPs) of these 5 protein-coding genes are associated with Behçet’s disease (BD). A two-stage association study was performed in a total of 907 BD patients and 1780 healthy controls. Altogether 32 polymorphisms were tested, using a Sequenom MassARRAY genotyping method in the first stage and a PCR-restriction fragment length polymorphism (PCR-RFLP) assay in the replication phase. Real-time PCR was performed to test the relative mRNA expression level of GAS6 and PROS1 from different SNP genotyped healthy individuals. The frequency of the C allele and CC genotype of rs9577873 in GAS6 (P_c_ = 4.92 × 10^−5^, P_c_ = 1.91 × 10^−5^, respectively) and A allele and AA genotype of rs4857037 in PROS1 (P_c_ = 1.85 × 10^−6^, P_c_ = 4.52 × 10^−7^, respectively) were significantly increased in BD. GAS6 expression in CC carriers of rs9577873 was significantly lower than that in CT/TT individuals (P = 0.001). Decreased expression of GAS6 and increased pro-inflammatory cytokines (IL-6 and IFN-γ: P = 4.23 × 10^−4^, P = 0.011, respectively) in individuals carrying the CC genotype suggest that the TAM-GAS6/PROS1 signal pathway may be involved in the pathogenesis of BD.

Uveitis is a spectrum of eye diseases with a highly complex etiology. It can be divided into several entities, depending on the presence of infectious pathogens and according to the clinical ocular manifestations, with or without typical extraocular features. Among them, Behçet’s disease (BD) is a common uveitis entity that mainly affects young adults in China^1^. BD is a chronic recurrent immune-disorder, clinically characterized by oral ulcers, genital ulcers, erythema nodosum, gastrointestinal tract lesions (ulceration and bowel perforation), articular lesions, relapsing vasculitis and nervous system abnormalities^2^, A higher prevalence of BD has been noted in individuals from a population along the “silk road” region that extends from the Far East to the Mediterranean sea area. Ocular involvement of BD is characterized by recurrent ocular inflammation (anterior and posterior uveitis and retinal vasculitis) and may cause visual loss within five years after disease onset. Unraveling the pathways involved during intraocular inflammation may lead to novel treatment opportunities that can hopefully prevent visual disability in these patients.

Although various studies indicate that environmental, immunological and genetic factors are involved in disease pathogenesis^3^, the precise etiopathogenesis has not yet been fully elucidated. Human Leukocyte Antigen (HLA) and non-HLA genes seem to jointly contribute to the genetic background leading to this disorder in different ethnic populations^4^. Many proteins involved in the immune response appear to be polymorphic and are considered to be genetic risk factors in the development of immune mediated diseases. HLA-B5/B51 and several non-HLA genes including TLR2^5^, TNFAIP3^6^, miR-182^7^, FAS^8^, IL23R-IL12RB2 and IL10^9^ are associated with BD. The Janus kinase (JAK) that belongs to a family of intracellular non-receptor tyrosine kinases, that transfer cytokine signals via the JAK-STAT pathway has also been identified as a risk factor for BD^10^,^11^,^12^. Other kinases may also play a role in BD development and this recently became a subject of interest in our laboratory.

The TAM receptors (TYRO3, AXL and Mer (gene name MERTK)) consist of a subfamily of receptor tyrosine kinases (RTK)^13^ that have two vitamin-K related agonists: GAS6 and protein S (ProS, PS; gene name PROS1)^14^,^15^,^16^. This small subfamily of RTKs has been implicated as pivotal effectors^17^ during immune modulation, including the regulation of innate immune reactions^18^,^19^,^20^, phagocytosis of apoptotic cells^21^,^22^,^23^, function and homeostasis of immune cells including dendritic cells^24^,^25^, monocytes/macrophages^26^, T cells^27^ and NK cells^28^,^29^, vascular integrity and even pathogenesis of cancer^30^. GAS6 has a variable potency to activate these 3 receptors (Axl>Tyro3>Mer) while ProS was specific to the latter two receptors, but does not interact with Axl^31^. Abnormal TAM signal components have been detected in the demyelination process of both experimental autoimmune encephalomyelitis (EAE) models^32^ and in multiple sclerosis (MS) patients^33^. Several SNPs within the MERTK gene have been identified from a genome-wide association study (GWAS) in MS in Australians and Europeans^33^. A mutation of MERTK has been reported to be associated with retinitis pigmentosa (RP) in the Royal College of Surgeons (RCS) rat and in humans because it compromised the phagocytic function of retinal pigment epithelial (RPE) cells^34^,^35^,^36^,^37^. A defective regulation of TAM receptors or their ligands has also been found in psoriasis^38^, primary Sjögren’s syndrome^39^ and inflammatory bowel disease (IBD)^40^. Previous studies also revealed that TAM-knockout mice easily develop systemic autoimmunity^41^,^42^. Appropriate apoptotic processes are essential to the development and maintenance of the immune system^43^. Abnormal clearance of apoptotic cells^22^,^44^ or resistance to cell death is believed to play a role in the pathogenesis and recurrences of autoimmune disorders such as rheumatoid arthritis (RA), systemic lupus erythematosus (SLE) and BD^8^,^45^. Significantly altered serum concentrations of soluble forms of TAM-GAS6/PS components have been found in BD^46^,^47^, SLE^48^, RA^49^ and IBD^50^ patients suggesting a potential role of TAM related signals in their pathogenesis.

Further support for a role of the TAM-GAS6/PS family in immune mediated disease comes from genetic association studies. Gene polymorphisms of the TAM family were associated with several immune-related disorders, including systemic lupus erythematosus^51^,^52^, type 2 diabetes^53^, diabetic nephropathy and systemic sclerosis^33^. In view of the given roles of these genes in immune disorders, we hypothesized that TYRO3, AXL, MERTK, GAS6 and PROS1 may also be associated with BD. Although abnormal expression of TAM-ligands in several tumor-associated or immune related diseases including BD^46^ and IBD^24^ have been reported, the relation between TAM-GAS6/PROS1 signal pathway gene polymorphisms with uveitis has not yet been reported in Han Chinese and was therefore the subject of the study presented here.

## Results

### Clinical feature of enrolled BD cases

Clinical and demographic information of the enrolled BD cases and controls (1154 male/ 626 female) are shown in Supplementary Table S1. Since the patients visited a department of Ophthalmology, the enrolled BD patients all had uveitis. Almost 85% of BD patients were male whereas 64.8% of controls were male.

### Genotype and allele frequencies of tested SNPs in cases and controls in the 1st, 2nd stage and combined studies

Thirty-two SNPs were genotyped in 412 BD and 612 healthy controls in the 1st stage study. The results showed significantly increased frequencies of the GAS6/rs9577873 C allele and CC genotype (P_c_ = 3.72 × 10^−2^, OR = 1.662; P_c_ = 2.42 × 10^−2^, OR = 1.772) and PROS1/rs4857037 A allele and AA genotype (P_c_ = 2.99 × 10^−3^, OR = 2.015; P_c_ = 2.37 × 10^−3^, OR = 2.117) (Tables 1 and 2). However, none of the remaining SNPs showed a significant association with ocular BD (Supplementary Table S2).

To validate the first stage study, another independent cohort including 495 BD patients and 1168 healthy controls were enrolled in the 2nd stage study. The results again showed significantly increased frequencies of the GAS6/rs9577873 C allele and CC genotype (P_c_ = 2.79 × 10^−2^, OR = 1.566; P_c_ = 1.51 × 10^−2^, OR = 1.666) and PROS1/rs4857037 A allele and AA genotype (P_c_ = 1.49 × 10^−2^, OR = 1.689; P_c_ = 4.80 × 10^−3^, OR = 1.825, respectively) in BD compared to controls. Combination of the data confirmed the association of rs9577873 (C allele: P_c_ = 4.92 × 10^−5^, OR = 1.598; CC genotype: P_c_ = 1.91 × 10^−5^, OR = 1.698)and rs4857037 (A allele: P_c_ = 1.85 × 10^−6^, OR = 1.822; AA genotype: P_c_ = 4.52 × 10^−7^, OR = 1.945) with BD (Tables 1 and 2).

A stratified analysis was performed to study whether an association of polymorphisms of GAS/rs9577873 and PROS1/rs4857037 was associated with some of the main clinical features of BD. However, no significant association was found for these SNP genotypes/alleles and 5 clinical manifestations in BD (Supplementary Table S3). Stratification for gender showed that genotype and allele frequency for both GAS6/rs9577873 and PROS1/rs4857037 showed a stronger significant difference in male (GAS6/rs9577873 C allele and CC genotype: P = 3.15 × 10^−6^, OR = 1.638; P =  2.55 × 10^−6^, OR = 1.720; PROS1/rs4857037 A allele and AA genotype: P = 4.16 × 10^−7^, OR = 1.840; P = 1.62 × 10^−7^, OR = 1.958) compared with female patients (Table 3).

Pairwise linkage disequilibrium (LD) and haplotype association analysis were performed using the SHEsis website. Six SNPs in the PROS1 gene (rs12634349-rs4857037-rs7616142-rs6803590-rs8178607-rs13062355) were in linkage disequilibrium with D’ ranging from 0.914 to 1.00 and r^2^ ranging from 0.009 to 0.707. Twelve SNPs in the GAS6 gene (rs9604488-rs7994900-rs7492052-rs6602910-rs12868833-rs7319547-rs7399860-rs9577924-rs7323932-rs9604466-rs9577873-rs7399637) were also in linkage disequilibrium with D’ ranging from 0.140 to 0.976 and r^2^ ranging from 0.005 to 0.682. The global haplotype frequencies were significantly different between the case and control group (P < 0.001). Furthermore, we investigated two kinds of PROS1 haplotypes (AATACA; AATGCG) that were more frequent in the case group than in the normal control groups, whereas the GATACA haplotype and AATACG haplotype were less frequent in the BD group than in the normal group (Supplementary Tables S4–8). There was no significant difference in the frequency distribution of the other haplotypes in these two groups.

### The effect of rs9577873 on GAS6 expression and cytokine production

**Figure 1:**
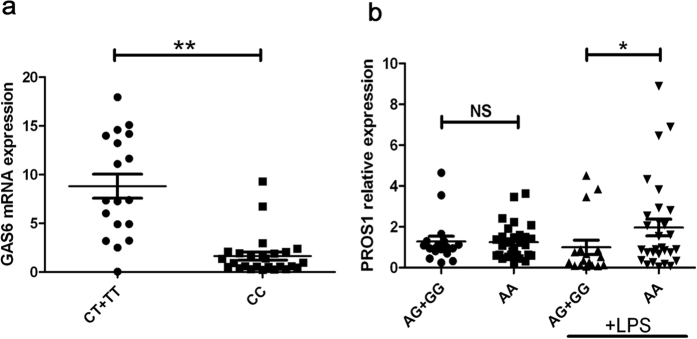
The effect of different genotypes of rs9577873 and rs4857037 on the expression of TAM ligand genes in PBMCs. (**a**) GAS6 expression in PBMCs from rs9577873 genotyped healthy controls (CC = 26, CT/TT = 21). GAS6 mRNA level in CC individuals of SNP rs9577873 was significantly lower than in CT/TT individuals. (**b**) PROS1 expression in PBMCs from healthy controls carrying different genotypes of rs4857037 (AA = 29–30 AG/GG = 16–17). PROS1 mRNA level in AA individuals of SNP rs4857037 was significantly higher than in AG/GG individuals when PBMCs were stimulated with LPS. Data are shown as mean ± SD. **P = 0.001 *P = 0.033.

**Figure 2:**
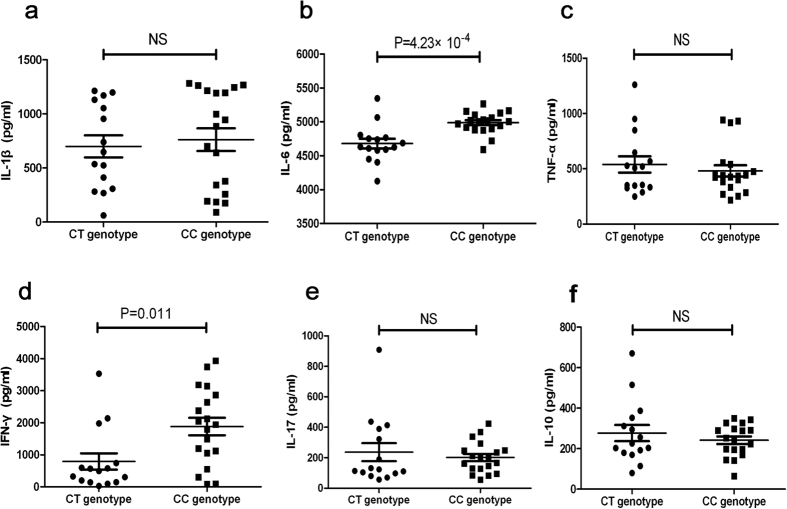
The effect of GAS6/rs9577873 genotypes on PBMC cytokine production. PBMCs were obtained from healthy rs9577873 genotyped controls (CC = 19, CT = 15–16) and were stimulated with LPS to investigate IL-1β, TNF-α and IL-6 production and with a cocktail of anti-CD3 and anti-CD28 antibodies to study IFN-γ, IL-17 and IL-10 production. An ELISA was used to detect IL-1β (**a**), IL-6 (**b**), TNF-α (**c**), IFN-γ (**d**), IL-17 (**e**) and IL-10 (**f**) in the cell culture supernatants. Data are shown as mean ± SD.

In order to find a possible functional association with GAS6/rs9577873, we carried out a real-time quantitative PCR assay to evaluate the GAS6 expression in PBMCs from known genotyped healthy individuals. We did not use patients for this functional analysis because inflammation and immunosuppressive drug use would confound the data. The results indicated that the GAS6 mRNA level in CC individuals of SNP rs9577873 was significantly lower compared with CT/TT individuals (Fig. 1a, P = 0.001). Several cytokines as well as Th1 and Th17 responses have been shown to play an important role in BD pathogenesis. GAS6 has been reported as an inhibitor of pro-inflammatory cytokines in monocytes/macrophages^26^. Further investigations were therefore performed to study whether various genotypes of rs9577873 altered cytokine production by PBMCs from healthy individuals. An elevated secretion of IL-6 (Fig. 2b, P = 4.23 × 10^−4^) and IFN-γ (Fig. 2d, P = 0.011) by stimulated PBMCs was observed in CC genotype when compared to CT genotype carriers. The different genotype carriers did not show differences concerning IL-1β, TNF-α, IL-17 or IL-10 production by stimulated PBMCs (Fig. 2a,c,e,f).

### The effect of rs4857037 on PROS1 expression and cytokine production

We also investigated the functional consequences of polymorphisms of rs4857037 in the PROS1 gene. No significant association in the expression of PROS1 by non-stimulated PBMCs from various rs4857037 genotype carriers could be detected (Fig. 1b). We also investigated whether the expression of PROS1 was affected by different rs4857037 genotypes in LPS-stimulated PBMCs. The PROS1 mRNA level in AA individuals of SNP rs4857037 was significantly higher than in AG/GG individuals when PBMCs had been stimulated with LPS (Fig. 1b, P = 0.033). Cytokine expression (IL-1β, IL-6, TNF-α, IL-10, IFN-γ and IL-17) by stimulated PBMCs was however not associated with PROS1/rs4857037 genotype (Supplementary Fig. S1a–f).

## Discussion

In the current study, we describe a novel association between receptor tyrosine kinase pathway (TAM-GAS6/PROS1) genes with ocular BD in a Chinese Han population. The observed association was restricted to the ligands GAS6 and PROS1 and no association could be detected with gene polymorphisms of the TAM receptors—TYRO3,AXL and MERTK. A significantly higher CC genotype and C allele carrier frequency of the GAS6/rs9577873 gene and AA genotype and A allele carrier frequency of the PROS1/rs4857037 gene was observed in BD. Functional analysis showed a significant down regulation of GAS6 mRNA expression by PBMCs in CC genotype compared with CT or TT carriers. PROS1/rs4857037 AA genotyped individuals showed an increased PROS1 expression compared to AG/GG carriers in LPS-stimulated PBMCs but not in non-stimulated cells. An elevated secretion of IL-6 and IFN-γ by LPS or anti-CD3 combined with anti-CD28 antibodies treated PBMCs was observed in CC genotype compared to CT genotype of GAS6/rs9577873 carriers. Stratification for gender showed that genotype and allele frequency for both GAS6/rs9577873 and PROS1/rs4857037 showed stronger significant differences in male compared with female patients. The observed SNP associations may thus be related via a regulation of gene transcription and modulation of inflammatory cytokine expression thereby leading to a higher risk of developing ocular BD.

TAMs belong to a family of tyrosine kinase receptors that have received little attention until recently, and were mostly related to TAM involvement in cancer^17^. Our study confirms earlier studies showing an association between the TAM-GAS6/PS pathway and immune related disorders such as psoriasis^38^, primary Sjögren’s syndrome^39^, SLE^48^, RA^49^ and IBD^40^,^50^. Studies on the role of gene polymorphisms in this pathway were limited to vascular and some autoimmune diseases including stroke^54^, type 2 diabetes^53^, SLE^51^,^52^ and systemic sclerosis^33^. Vasculitis is a prominent feature of BD and it is possible that the role of TAM-GAS6/PS in this disease may be related to its involvement in the control of vascular integrity^17^. The TAM-GAS6/PS signal pathway has been shown to play a pivotal role in blocking the innate immune response and has also been associated with the regulation of cancer development^17^. The TAM-GAS6/PS system is widely expressed in a variety of immune cells, and has been shown to play a role in the EAE^32^ model and in retinitis pigmentosa^35^. TAM-GAS6/PS also plays critical roles in the maintenance of retinal function via its effect on retinal pigment epithelial phagocytosis and altering the expression of microRNAs^37^,^55^. It has also been shown to play a role in a mouse model of human uveitis known as experimental autoimmune uveoretinitis (EAU), which can be induced by immunization of the experimental animals with retinal autoantigens, such as interphotoreceptor retinoid- binding protein (IRBP). It has been reported that TAM receptor knockout mice were more susceptible to IRBP immunization supporting its negative control of the inflammatory process^42^. Our findings are in agreement with these previous studies showing that suppression of the TAM signal or knocking-out TAM in mice makes them more prone to autoimmunity^41^,^42^.

Apoptosis is essential to the development and maintenance of the immune system^56^ and the TAM-GAS6/PS system has been shown to mainly exert its role in immunity by its effect on the phagocytosis of apoptotic cells^21^,^23^,^25^ and thereby maintaining immune homeostasis^14^,^18^. Changes in the plasma concentration of Axl/Gas6 have been reported in rheumatic diseases such as SLE^48^, BD^46^,^47^ and RA^49^. Significantly increased Axl levels were also observed in patients with BD^47^. This may be caused by a dysfunctional feedback or interaction of the TAM-GAS6/PS system resulting in an elevated Axl level. There is a growing body of evidence suggesting that chronic inflammation in different autoimmune diseases such as BD^45^,^57^, RA^58^ and multiple sclerosis is caused by the apoptosis refractory nature of activated T cells and a continuous stimulation caused by apoptotic debris^21^. The abnormal apoptotic process seems critical for autoimmune disease pathogenesis and may be involved in causing the recurrent and chronic character of the disease. Collagen-induced arthritis (CIA) is used as an animal model for RA and several studies have shown that agonists of the TAM pathway may be used to block inflammation in this model^59^.

Earlier GWAS approaches in BD did not reveal an association with the TAM-GAS6/PS pathway. The reason for this discrepancy may be explained as follows. Although GWAS is a powerful approach to scan and find complicated disease related loci, it is based on the assumption of indirect association mapping using reference linkage SNPs and has a strict P value set at the 10^−8^ level. Its result may vary according to ethnicity, sample sources, sample size and GWAS chip coverage in the discovery phase. For example, the Affymetrix 500k chip (Affymetrix Company, Santa Clara, CA, USA) can capture about 65% of the common variants and the Ilumina 317k chips (Illumina Company, San Diego, CA, USA) can capture about 75% of the common variants. GWAS therefore does not cover all possible common variants. Our study used a p value with a lower threshold as used for GWAS and may therefore have picked up loci not detected by GWAS.

Previous findings suggested that TAM-ligands may be associated with BD activity especially in neuro-BD, where the serum GAS6 level was shown to be decreased in BD^47^. Although the consequences of diminished mRNA levels of GAS6 in CC genotype carriers is not clear, it may suggest an involvement of the TAM-GAS6/PS signal pathway during abnormal apoptotic processes^21^ in patients with BD. Further experiments investigating apoptotic function and hnRNA expression in relation to TAM polymorphisms are necessary to clarify this subject. Further confirmation of the role of this pathway in BD pathogenesis is necessary and this may involve the measurement of TAM-GAS6/PS related products in the blood of our BD patients. It is also not clear whether the observed association is confined to BD patients with uveitis or whether it can also be observed in other uveitis entities. Since BD is a multisystemic disease, it would also be interesting to investigate whether the observed association is restricted to ocular BD. Future investigations are therefore needed in a large cohort of BD patients recruited via other medical departments including rheumatology and dermatology to address this issue. Our study was performed in a Han Chinese population and verification studies are needed in other ethnic populations. Our study does not exclude the possibility that other SNPs or copy number variants (CNVs) of TAM and its ligand genes are associated with this disease. Additional SNPs around the tagging SNP should be genotyped to identify the underlying haplotype. Further investigations are needed to address these questions and studies concerning specific agonists or analogues targeting this pathway may offer therapeutic opportunities in the near future.

In conclusion, our results show that polymorphisms in the genes encoding the ligands of the receptor tyrosine kinase family, GAS6 and PROS1 confer genetic susceptibility for ocular BD in Han Chinese.

## Materials and Methods

### Study participant recruitment

A total of 412 patients who fulfilled the criteria for Behçet’t Disease according to the International Study Group diagnostic criteria^60^ were included in the first phase study. Six hundred and twelve age, geographically and ethnically matched healthy Chinese Han volunteers served as controls. Another set of 495 BD patients and 1168 healthy controls were included in the replication study. They were recruited consecutively by the Ophthalmology department of the First Affiliated Hospital of Chongqing Medical University (Chongqing, P.R. China) from May 2008 to August 2015.

### Ethical considerations

The experimental protocols and study design were approved by the local ethical research committee of the First Affiliated Hospital of Chongqing Medical University. All experiments were carried out in accordance with the approved guidelines. The ethical standards of the Declaration of Helsinki were followed during all the experimental procedures. All study participants were well informed and signed an informed consent before their enrollment.

### Tag SNP selection

The choice of SNPs was mainly based on tagSNPs. Thirty-two tagSNPs involving 5 TAM signal genes were chosen in the present study. After a search in the public database HapMap and HaploView (V4.0; Daly lab at the Broad Institute, Cambridge, MA, USA) and specific analysis for the Han Chinese in Beijing (CHB) population, our candidate tagSNPs were chosen based on a minor allele frequency (MAF) >0.05 and r^2^ was set at 0.8. We chose a total of thirty-two SNPs: two in AXL, one in TYRO3, eleven in MERTK, twelve in GAS6 and six in PROS1.

### Genomic DNA preparation and SNP genotyping analysis

Peripheral whole blood samples of patients and healthy volunteers were collected into EDTA containing tubes by venipuncture. Genomic DNA was extracted from peripheral blood using the commercial QIAamp DNA Blood Mini Kit (Qiagen, Valencia, California, USA) according to the manufacturer’s protocols. All the isolated DNA samples were quantified with a Nanodrop 2000 (Thermo Fisher Scientific, Wilmington, DE, USA), quality checked, standardized and stored at −20 °C until assayed. The primers used for genotyping were designed by MassARRAY Assay design software. SNP genotyping in the discovery cohort was determined using the Sequenom MassARRAY system platform (Sequenom Inc, San Diego, California, USA) and iPLEX reagents according to the manufacturer’s instructions (Agena Bioscience, California, USA). The PCR reaction was performed on the GeneAmp PCR System 9700 instrument (ABI, Foster City, CA, USA). Subjects in the replication phase were genotyped using the PCR-RFLP method. Appropriate primers and restriction enzymes (Table 4) were used to amplify the target DNA sequence and digest the PCR product respectively. After digesting the PCR products with restriction enzymes at 37 °C, the digested products were subjected to electrophoresis using 4% or 5% agarose gels, stained by GoldView (SBS Genetech, Beijing, China) and visualized under a Bio-Rad imaging system. Randomly selected samples (3%) were checked using direct sequencing by Sangon Biotech Company (Sangon Biotech, Shanghai, China) and the results were in complete accordance with the Sequenom MassARRAY system genotyping and PCR-RFLP outcomes.

### Cell isolation and culture

Fresh peripheral blood from healthy volunteers was used to isolate peripheral blood mononuclear cells (PBMCs) by Ficoll-Hypaque density gradient centrifugation. A density of 1 × 10^6^ PBMCs per well were seeded into 24-well culture plates with complete medium RPMI 1640 (containing 10%FBS, 100 U/mL penicillin and 100 μg/mL streptomycin) and stimulated with 100 ng/mL lipopolysaccharide (LPS)(Sigma, Missouri, USA) for 24 h to detect IL-1β, TNF-α and IL-6 production. Antigen stimulation was simulated using a cocktail of anti-CD3 and anti-CD28 antibodies (5:1) (Miltenyi Biotec, Palo Alto, CA) for 3 days at 37 °C in a humidified 5% CO_2_ incubator where after IL-10, IL-17 and IFN-γ were measured in the culture supernatants.

### RNA preparation, reverse transcription and Real-time PCR

Total RNA was extracted from LPS-stimulated PBMCs and non-stimulated PBMCs using the TRIzol (Invitrogen, San Diego, California, USA) method. Reverse transcription was then performed using a Takara transcriptase kit (Takara, Dalian, China). Relative mRNA expression assays were performed on an ABI 7500 real-time instrument. The expression of GAS6 and PROS1 mRNA relative to β-actin was calculated using the 2^−ΔΔCt^ method. The GAS6 and PROS1 expression was detected using primers reported elsewhere^39^. All samples were evaluated in duplicate with at least three experimental replicates.

### Cytokine ELISAs

The concentration of IL-1β, TNF-α, IL-6, IL-10, IL-17 and IFN-γ in the culture supernatants of LPS or anti-CD3 combined with anti-CD28 antibodies treated PBMCs was measured using the human Duoset ELISA development kit (R&D Systems, Minneapolis, Minnesota, USA). Recombinant proteins were used to generate the standard curve. Test results were evaluated using an ELISA reader (SpectraMax M2e, Molecular Devices, USA) at 450 nm.

### Statistical analysis

Genotype and allele frequency data were calculated by Typer4.0 software from the MassARRAY system platform or by a direct count from the PCR-RFLP result. The SHEsis website was used to test the Hardy-Weinberg equilibrium (HWE) in controls of all tested SNPs. No HWE deviation was observed. Pairwise linkage disequilibrium (LD) and haplotype association analysis were also performed using the SHEsis website. SPSS (SPSS Inc., Chicago, Illinois, USA) version 17.0 was used to analyze the χ^2^ test, P value, odds ratio (OR) as well as 95% confidence intervals (95% CIs) data. To correct for multiple comparisons, the P values were adjusted as corrected P values (P_c_) with the Bonferroni correction approach according to the number of analyses performed. Statistical significance level was set at P_c_ < 0.05. Continuous variables were summarized through the mean ± SD. Student t test or the Nonparametric Mann-Whitney U test was selected for independent group comparisons. P values of 0.05 or less (Two-tailed) were considered as statistically significant.

## Additional Information

**How to cite this article**: Qin, J. *et al*. Analysis of receptor tyrosine kinase genetics identifies two novel risk loci in GAS6 and PROS1 in Behçet’s disease. *Sci. Rep*. **6**, 26662; doi: 10.1038/srep26662 (2016).

## Supplementary Material

Supplementary Information

## Figures and Tables

**Table 1:** Summary of SNPs for TAM-GAS6/PROS1 allele frequencies with Behcet’s Disease in the First Stage.

Gene	SNP	Allele	Case	(freq.)	Control	(freq.)	P value	P_c_ value	OR(95% CI)
AXL	rs1051008	C	771	(0.936)	1144	(0.935)	0.925	NS	1.017(0.710–1.457)
	rs11882467	G	517	(0.629)	789	(0.647)	0.412	NS	0.926(0.770–1.113)
TYRO3	rs2277537	C	681	(0.828)	1025	(0.837)	0.594	NS	0.938(0.740–1.188)
MERTK	rs10199083	C	653	(0.8)	969	(0.792)	0.638	NS	1.054(0.846–1.314)
	rs11674891	A	743	(0.902)	1082	(0.884)	0.207	NS	1.204(0.902–1.606)
	rs11884641	A	731	(0.887)	1100	(0.899)	0.405	NS	0.886(0.667–1.178)
	rs11887259	T	656	(0.796)	949	(0.775)	0.262	NS	1.132(0.910–1.405)
	rs12477716	C	750	(0.912)	1115	(0.911)	0.909	NS	1.018(0.746–1.391)
	rs4848958	T	739	(0.897)	1064	(0.869)	0.059	NS	1.307(0.989–1.729)
	rs6738237	A	757	(0.919)	1143	(0.935)	0.151	NS	0.781(0.557–1.095)
	rs7569614	T	620	(0.752)	886	(0.724)	0.151	NS	1.159(0.948–1.419)
	rs7580261	C	633	(0.768)	957	(0.782)	0.467	NS	0.925(0.749–1.142)
	rs867311	G	771	(0.936)	1099	(0.936)	0.969	NS	0.993(0.690–1.428)
	rs869016	T	650	(0.791)	935	(0.764)	0.154	NS	1.168(0.943–1.446)
GAS6	rs12868833	G	790	(0.959)	1186	(0.969)	0.218	NS	0.744(0.465–1.193)
	rs6602910	A	482	(0.585)	675	(0.551)	0.134	NS	1.146(0.959–1.370)
	rs7319547	A	728	(0.883)	1076	(0.879)	0.763	NS	1.043(0.793–1.371)
	rs7323932	T	614	(0.745)	914	(0.747)	0.936	NS	0.992(0.810–1.215)
	rs7399637	G	550	(0.694)	827	(0.676)	0.376	NS	1.091(0.900–1.323)
	rs7399860	A	556	(0.678)	823	(0.676)	0.911	NS	1.011(0.836–1.222)
	rs7492052	G	704	(0.856)	1037	(0.847)	0.566	NS	1.076(0.838–1.381)
	rs7994900	G	575	(0.698)	847	(0.692)	0.779	NS	1.028(0.848–1.245)
	rs9577873	C	747	(0.907)	1045	(0.854)	3.96 × 10^−4^	3.72 × 10^−2^	1.662(1.252–2.206)
	rs9577924	T	660	(0.801)	928	(0.759)	0.027	NS	1.275(1.028–1.582)
	rs9604466	A	678	(0.823)	998	(0.817)	0.724	NS	1.042(0.828–1.312)
	rs9604488	G	457	(0.555)	635	(0.519)	0.111	NS	1.155(0.967–1.379)
PROS1	rs12634349	G	484	(0.587)	709	(0.579)	0.714	NS	1.034(0.864–1.237)
	rs13062355	A	529	(0.644)	780	(0.637)	0.771	NS	1.028(0.855–1.236)
	rs4857037	A	774	(0.939)	1083	(0.885)	3.18 × 10^−5^	2.99 × 10^−3^	2.015(1.441–2.819)
	rs6803590	A	620	(0.752)	945	(0.772)	0.305	NS	0.897(0.729–1.104)
	rs7616142	T	785	(0.953)	1164	(0.951)	0.861	NS	1.038(0.686–1.568)
	rs8178607	C	748	(0.908)	1083	(0.892)	0.25	NS	1.191(0.884–1.603)

P_c_ value: the Bonferroni corrected P value; NS: not significant.

**Table 2:** Detailed information about Genotype/Allele of SNPs for GAS6/PROS1 Gene with Behcet Disease in the First and Replication Stage.

SNP	Stage	Genotype/Allele	Case	(freq.)	Control	(freq.)	P value	P_c_ value	OR(95% CI)
rs9577873	1st stage	CC	339	(0.823)	443	(0.724)	2.57 × 10^−4^	2.42 × 10^−2^	1.772(1.301–2.413)
		CT	69	(0.167)	159	(0.26)	4.97 × 10^−4^	4.67 × 10^−2^	0.573(0.418–0.786)
		TT	4	(0.01)	10	(0.016)	0.37	NS	0.590(0.184–1.895)
		C	747	(0.907)	1045	(0.854)	3.96 × 10^−4^	3.72 × 10^−2^	1.662(1.252–2.206)
	2nd stage	CC	409	(0.826)	865	(0.741)	1.61 × 10^−4^	1.51 × 10^−2^	1.666(1.276–2.175)
		CT	80	(0.162)	283	(0.242)	2.71 × 10^−4^	2.55 × 10^−2^	0.603(0.458–0.793)
		TT	6	(0.012)	20	(0.017)	0.452	NS	0.704(0.281–1.765)
		C	898	(0.907)	2013	(0.862)	2.97 × 10^−4^	2.79 × 10^−2^	1.566(1.226–2.000)
	combined	CC	748	(0.825)	1308	(0.735)	2.03 × 10^−7^	1.91 × 10^−5^	1.698(1.389–2.075)
		CT	149	(0.164)	442	(0.248)	6.59 × 10^−7^	6.19 × 10^−5^	0.595(0.484–0.731)
		TT	10	(0.011)	30	(0.017)	0.238	NS	0.650(0.316–1.336)
		C	1645	(0.907)	3058	(0.859)	5.23 × 10^−7^	4.92 × 10^−5^	1.598(1.329–1.921)
rs4857037	1st stage	AA	363	(0.881)	476	(0.778)	2.52 × 10^−5^	2.37 × 10^−3^	2.117(1.486–3.016)
		AG	48	(0.117)	131	(0.214)	5.57 × 10^−5^	5.24 × 10^−3^	0.484(0.339–0.692)
		GG	1	(0.002)	5	(0.008)	0.238	NS	0.295(0.034–2.537)
		A	774	(0.939)	1083	(0.885)	3.18 × 10^−5^	2.99 × 10^−3^	2.015(1.441–2.819)
	2nd stage	AA	429	(0.867)	912	(0.781)	5.10 × 10^−5^	4.80 × 10^−3^	1.825(1.360–2.448)
		AG	62	(0.125)	246	(0.211)	4.18 × 10^−5^	3.93 × 10^−3^	0.537(0.397–0.725)
		GG	4	(0.008)	10	(0.009)	0.922	NS	0.943(0.294–3.022)
		A	920	(0.929)	2070	(0.886)	1.59 × 10^−4^	1.49 × 10^−2^	1.689(1.283–2.222)
	combined	AA	792	(0.873)	1388	(0.78)	4.81 × 10^−9^	4.52 × 10^−7^	1.945(1.552–2.437)
		AG	110	(0.121)	377	(0.212)	8.42 × 10^−9^	7.91 × 10^−7^	0.514(0.408–0.646)
		GG	5	(0.006)	15	(0.008)	0.406	NS	0.652(0.236–1.800)
		A	1694	(0.934)	3153	(0.886)	1.96 × 10^−8^	1.85 × 10^−6^	1.822(1.474–2.253)

P_c_ value: the Bonferroni correction P value; NS: not significant.

**Table 3:** Polymorphisms of GAS/rs9577873 and PROS1/rs4857037 according to gender in BD.

SNP	Male					Female			
	Genotype/Allele	BD n = 770	Control n = 1154	P value	OR(95% CI)	BD n = 137	Control n = 626	P value	OR(95% CI)
rs9577873	CC	636	847	2.55 × 10^−6^	1.720(1.370–2.160)	112	461	0.047	1.603(1.004–2.562)
	CT	127	287	1.18 × 10^−5^	0.597(0.473–0.753)	22	155	0.029	0.581(0.356–0.950)
	TT	7	20	0.132	0.520(0.219–1.236)	3	10	0.627	1.379(0.374–5.079)
	C	1399	1981	3.15 × 10^−6^	1.638(1.329–2.018)	246	1077	0.097	1.428(0.936–2.178)
rs4857037	AA	673	900	1.62 × 10^−7^	1.958(1.518–2.525)	119	488	0.019	1.870(1.100–3.178)
	AG	93	244	2.96 × 10^−7^	0.512(0.396–0.664)	17	133	0.018	0.525(0.305–0.904)
	GG	4	10	0.38	0.597(0.187–1.912)	1	5	0.934	0.913(0.106–7.880)
	A	1439	2044	4.16 × 10^−7^	1.840(1.449–2.337)	255	1109	0.029	1.731(1.052–2.846)

**Table 4:** Gene location, Primers and Restriction Enzymes used for PCR-RFLP in the Replication Stage.

Chromosome Location	Gene	SNP	Primers	Restriction Enzyme
13q34	GAS6	rs9577873	Forward 5′-TACTGGCCTGGCTCACTCT-3′	XbaI
			Reverse 5′-GGAAGCTCCTGACAGGAGTCTAG-3′	
3q11.2	PROS1	rs4857037	Forward 5′-GAGTCACAGTGTTCTGCT-3′	AccI
			Reverse 5′-AGGCACATATCATCACTCCT-3′	
