# Supplementary material for: Analysis of receptor tyrosine kinase genetics identifies two novel risk loci in GAS6 and PROS1 in Behçet’s disease
Source: Sci Rep. 2016 May 25;6:26662. doi: 10.1038/srep26662 (PMC4879572; doi:10.1038/srep26662)
Supplement: Supplementary Information [file srep26662-s1.doc]

**Analysis of receptor tyrosine kinase genetics identifies two novel risk loci in GAS6 and PROS1 in Behçet disease**

Jieying Qin^1^,*,Lin Li^1^,*,Donglei Zhang^1^,Hongsong Yu^1^,Handan Tan^1^,Jun Zhang^1^,Bolin Deng^1^,Aize Kijlstra^2^ ,Peizeng Yang^1^

1The First Affiliated Hospital of Chongqing Medical University, Chongqing Key Laboratory of Ophthalmology and Chongqing Eye Institute, Chongqing, People’s Republic of China

2University Eye Clinic Maastricht, Maastricht, The Netherlands

*These authors contributed equally to this work.

**Supplementary figure**

**
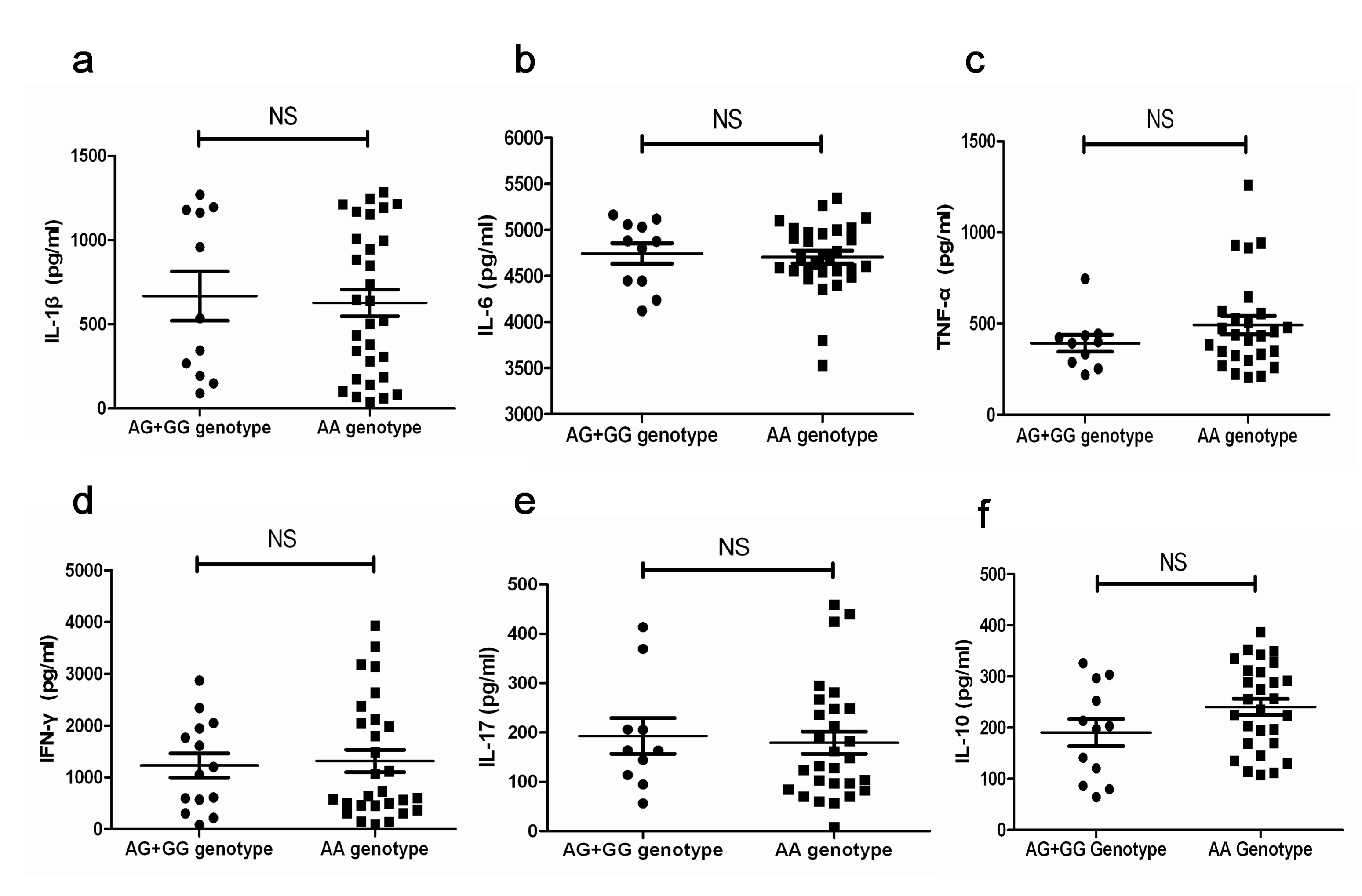
**

**Figure S1**.The effect of PROS1/rs4857037 on PBMC cytokine production. The production of IL-1β(a), IL-6(b), TNF-α(c), IFN-γ(d), IL-17(e) and IL-10(f) by stimulated PBMCs from healthy controls carrying different genotypes of rs4857037(AA=28-30,AG/GG=11-12) was investigated following the same procedure as outlined in figure 2. Data are shown as mean±SD.

**Supplementary tables**

**Table S1** Clinical Features of ocular Behcet’s Disease Patients and Controls enrolled in the Study

| **Clinical features** | **Number** | **Percentage (%)** |
| --- | --- | --- |
| Total | 907 |  |
| Mean Age±SD | 35.7±8.7 |  |
| Male | 770 | 84.90 |
| Female | 137 | 15.10 |
| Uveitis | 907 | 100.00 |
| Arthritis | 135 | 14.88 |
| Hypopyon | 233 | 25.69 |
| Genital ulcer | 494 | 54.47 |
| Oral ulcer | 907 | 100.00 |
| Positive pathergy test | 192 | 21.17 |
| Skin lesions | 648 | 71.44 |
| **Controls** | 1780 |  |
| Mean Age±SD | 39.4±10.6 |  |
| Male | 1154 | 64.83 |
| Female | 626 | 35.17 |

**Table S2** Detailed information about Genotype/Allele frequency of SNPs for TAM-GAS6/PROS1 Gene with Behcet’s Disease in the First Stage

| Gene | SNP | Genotype | Case | (freq.) | Control | (freq.) | P value | P_c_ value | OR(95%CI) |
| --- | --- | --- | --- | --- | --- | --- | --- | --- | --- |
|  |  | /Allele |  |  |  |  |  |  |  |
| AXL | rs1051008 | CC | 360 | (0.874) | 535 | (0.874) | 0.985 | NS | 0.996(0.684-1.452) |
|  |  | CT | 51 | (0.124) | 74 | (0.121) | 0.891 | NS | 1.027(0.702-1.503) |
|  |  | TT | 1 | (0.002) | 3 | (0.005) | 0.534 | NS | 0.494(0.051-4.765) |
|  |  | C | 771 | (0.936) | 1144 | (0.935) | 0.925 | NS | 1.017(0.710-1.457) |
|  | rs11882467 | GG | 166 | (0.404) | 258 | (0.423) | 0.544 | NS | 0.924(0.717-1.192) |
|  |  | GT | 185 | (0.45) | 273 | (0.448) | 0.935 | NS | 1.010(0.786-1.299) |
|  |  | TT | 60 | (0.146) | 79 | (0.13) | 0.452 | NS | 1.149(0.800-1.650) |
|  |  | G | 517 | (0.629) | 789 | (0.647) | 0.412 | NS | 0.926(0.770-1.113) |
| TYRO3 | rs2277537 | CC | 286 | (0.696) | 431 | (0.704) | 0.774 | NS | 0.961(0.732-1.262) |
|  |  | CT | 109 | (0.265) | 163 | (0.266) | 0.968 | NS | 0.994(0.749-1.319) |
|  |  | TT | 16 | (0.039) | 18 | (0.029) | 0.405 | NS | 1.337(0.674-2.653) |
|  |  | C | 681 | (0.828) | 1025 | (0.837) | 0.594 | NS | 0.938(0.740-1.188) |
| MERTK | rs10199083 | CC | 263 | (0.645) | 382 | (0.624) | 0.507 | NS | 1.092(0.842-1.417) |
|  |  | CT | 127 | (0.311) | 205 | (0.335) | 0.429 | NS | 0.897(0.686-1.174) |
|  |  | TT | 18 | (0.044) | 25 | (0.041) | 0.799 | NS | 1.084(0.583-2.013) |
|  |  | C | 653 | (0.8) | 969 | (0.792) | 0.638 | NS | 1.054(0.846-1.314) |
|  | rs11674891 | AA | 334 | (0.811) | 477 | (0.779) | 0.227 | NS | 1.212(0.887-1.655) |
|  |  | AG | 75 | (0.182) | 128 | (0.209) | 0.286 | NS | 0.842(0.613-1.156) |
|  |  | GG | 3 | (0.007) | 7 | (0.011) | 0.507 | NS | 0.634(0.163-2.466) |
|  |  | A | 743 | (0.902) | 1082 | (0.884) | 0.207 | NS | 1.204(0.902-1.606) |
|  | rs11884641 | AA | 324 | (0.786) | 491 | (0.802) | 0.536 | NS | 0.907(0.667-1.235) |
|  |  | AG | 83 | (0.201) | 118 | (0.193) | 0.733 | NS | 1.056(0.772-1.445) |
|  |  | GG | 5 | (0.012) | 3 | (0.005) | 0.197 | NS | 2.494(0.593-10.493) |
|  |  | A | 731 | (0.887) | 1100 | (0.899) | 0.405 | NS | 0.886(0.667-1.178) |
|  | rs11887259 | TT | 263 | (0.638) | 363 | (0.593) | 0.146 | NS | 1.211(0.936-1.567) |
|  |  | TC | 130 | (0.316) | 223 | (0.364) | 0.107 | NS | 0.804(0.617-1.048) |
|  |  | CC | 19 | (0.046) | 26 | (0.042) | 0.781 | NS | 1.090(0.595-1.996) |
|  |  | T | 656 | (0.796) | 949 | (0.775) | 0.262 | NS | 1.132(0.910-1.405) |
|  | rs12477716 | CC | 342 | (0.832) | 505 | (0.825) | 0.773 | NS | 1.050(0.753-1.464) |
|  |  | CT | 66 | (0.161) | 105 | (0.172) | 0.644 | NS | 0.924(0.660-1.294) |
|  |  | TT | 3 | (0.007) | 2 | (0.003) | 0.365 | NS | 2.243(0.373-13.480) |
|  |  | C | 750 | (0.912) | 1115 | (0.911) | 0.909 | NS | 1.018(0.746-1.391) |
|  | rs4848958 | TT | 332 | (0.806) | 460 | (0.752) | 0.042 | NS | 1.371(1.011-1.861) |
|  |  | TC | 75 | (0.182) | 144 | (0.235) | 0.042 | NS | 0.723(0.529-0.988) |
|  |  | CC | 5 | (0.012) | 8 | (0.013) | 0.896 | NS | 0.928(0.301-2.855) |
|  |  | T | 739 | (0.897) | 1064 | (0.869) | 0.059 | NS | 1.307(0.989-1.729) |
|  | rs6738237 | AA | 347 | (0.842) | 534 | (0.874) | 0.15 | NS | 0.770(0.539-1.100) |
|  |  | AG | 63 | (0.153) | 75 | (0.123) | 0.166 | NS | 1.290(0.899-1.851) |
|  |  | GG | 2 | (0.005) | 2 | (0.003) | 0.691 | NS | 1.485(0.208-10.587) |
|  |  | A | 757 | (0.919) | 1143 | (0.935) | 0.151 | NS | 0.781(0.557-1.095) |
|  | rs7569614 | TT | 234 | (0.568) | 315 | (0.515) | 0.094 | NS | 1.239(0.964-1.594) |
|  |  | CT | 152 | (0.369) | 256 | (0.418) | 0.114 | NS | 0.813(0.629-1.051) |
|  |  | CC | 26 | (0.063) | 41 | (0.067) | 0.805 | NS | 0.938(0.564-1.559) |
|  |  | T | 620 | (0.752) | 886 | (0.724) | 0.151 | NS | 1.159(0.948-1.419) |
|  | rs7580261 | CC | 241 | (0.585) | 372 | (0.608) | 0.464 | NS | 0.909(0.705-1.173) |
|  |  | CT | 151 | (0.367) | 213 | (0.348) | 0.545 | NS | 1.084(0.835-1.406) |
|  |  | TT | 20 | (0.049) | 27 | (0.044) | 0.74 | NS | 1.105(0.611-1.999) |
|  |  | C | 633 | (0.768) | 957 | (0.782) | 0.467 | NS | 0.925(0.749-1.142) |
|  | rs867311 | GG | 362 | (0.879) | 515 | (0.877) | 0.951 | NS | 1.012(0.689-1.488) |
|  |  | GT | 47 | (0.114) | 69 | (0.118) | 0.866 | NS | 0.967(0.652-1.434) |
|  |  | TT | 3 | (0.007) | 3 | (0.005) | 0.662 | NS | 1.428(0.287-7.110) |
|  |  | G | 771 | (0.936) | 1099 | (0.936) | 0.969 | NS | 0.993(0.690-1.428) |
|  | rs869016 | TT | 257 | (0.625) | 350 | (0.572) | 0.088 | NS | 1.249(0.967-1.614) |
|  |  | TC | 136 | (0.331) | 235 | (0.384) | 0.083 | NS | 0.793(0.610-1.031) |
|  |  | CC | 18 | (0.044) | 27 | (0.044) | 0.98 | NS | 0.992(0.539-1.826) |
|  |  | T | 650 | (0.791) | 935 | (0.764) | 0.154 | NS | 1.168(0.943-1.446) |
| GAS6 | rs12868833 | GG | 380 | (0.922) | 574 | (0.938) | 0.333 | NS | 0.786(0.483-1.280) |
|  |  | GA | 30 | (0.073) | 38 | (0.062) | 0.499 | NS | 1.186(0.722-1.948) |
|  |  | AA | 2 | (0.005) | 0 | (0) | / | / |  |
|  |  | G | 790 | (0.959) | 1186 | (0.969) | 0.218 | NS | 0.744(0.465-1.193) |
|  | rs6602910 | AA | 144 | (0.35) | 187 | (0.306) | 0.14 | NS | 1.221(0.936-1.593) |
|  |  | AG | 194 | (0.471) | 301 | (0.492) | 0.511 | NS | 0.919(0.716-1.181) |
|  |  | GG | 74 | (0.18) | 124 | (0.203) | 0.361 | NS | 0.862(0.626-1.186) |
|  |  | A | 482 | (0.585) | 675 | (0.551) | 0.134 | NS | 1.146(0.959-1.370) |
|  | rs7319547 | AA | 326 | (0.791) | 477 | (0.779) | 0.651 | NS | 1.073(0.791-1.455) |
|  |  | AG | 76 | (0.184) | 122 | (0.199) | 0.554 | NS | 0.908(0.661-1.249) |
|  |  | GG | 10 | (0.024) | 13 | (0.021) | 0.538 | NS | 0.784(0.361-1.704) |
|  |  | A | 728 | (0.883) | 1076 | (0.879) | 0.763 | NS | 1.043(0.793-1.371) |
|  | rs7323932 | TT | 228 | (0.553) | 345 | (0.564) | 0.744 | NS | 0.959(0.746-1.233) |
|  |  | TC | 158 | (0.383) | 224 | (0.366) | 0.571 | NS | 1.077(0.833-1.394) |
|  |  | CC | 26 | (0.063) | 43 | (0.07) | 0.654 | NS | 0.891(0.539-1.475) |
|  |  | T | 614 | (0.745) | 914 | (0.747) | 0.936 | NS | 0.992(0.810-1.215) |
|  | rs7399637 | GG | 197 | (0.497) | 285 | (0.466) | 0.324 | NS | 1.136(0.882-1.463) |
|  |  | GA | 156 | (0.394) | 257 | (0.42) | 0.412 | NS | 0.898(0.694-1.162) |
|  |  | AA | 43 | (0.109) | 70 | (0.114) | 0.776 | NS | 0.943(0.631-1.411) |
|  |  | G | 550 | (0.694) | 827 | (0.676) | 0.376 | NS | 1.091(0.900-1.323) |
|  | rs7399860 | AA | 195 | (0.476) | 288 | (0.473) | 0.932 | NS | 1.011(0.787-1.299) |
|  |  | AC | 166 | (0.405) | 247 | (0.406) | 0.982 | NS | 0.997(0.773-1.287) |
|  |  | CC | 49 | (0.12) | 74 | (0.122) | 0.124 | NS | 1.357(0.919-2.005) |
|  |  | A | 556 | (0.678) | 823 | (0.676) | 0.911 | NS | 1.011(0.836-1.222) |
|  | rs7492052 | GG | 299 | (0.727) | 441 | (0.721) | 0.809 | NS | 1.035(0.782-1.369) |
|  |  | GA | 106 | (0.258) | 155 | (0.253) | 0.867 | NS | 1.025(0.770-1.364) |
|  |  | AA | 6 | (0.015) | 16 | (0.026) | 0.212 | NS | 0.552(0.214-1.422) |
|  |  | G | 704 | (0.856) | 1037 | (0.847) | 0.566 | NS | 1.076(0.838-1.381) |
|  | rs7994900 | GG | 198 | (0.481) | 295 | (0.482) | 0.964 | NS | 0.994(0.774-1.277) |
|  |  | GA | 179 | (0.434) | 257 | (0.42) | 0.645 | NS | 1.061(0.824-1.366) |
|  |  | AA | 35 | (0.085) | 60 | (0.098) | 0.479 | NS | 0.854(0.552-1.322) |
|  |  | G | 575 | (0.698) | 847 | (0.692) | 0.779 | NS | 1.028(0.848-1.245) |
|  | rs9577873 | CC | 339 | (0.823) | 443 | (0.724) | 2.57×10^-4^ | 2.42×10^-2^ | 1.772(1.301-2.413) |
|  |  | CT | 69 | (0.167) | 159 | (0.26) | 4.97×10^-4^ | 4.67×10^-2^ | 0.573(0.418-0.786) |
|  |  | TT | 4 | (0.01) | 10 | (0.016) | 0.37 | NS | 0.590(0.184-1.895) |
|  |  | C | 747 | (0.907) | 1045 | (0.854) | 3.96×10^-4^ | 3.72×10^-2^ | 1.662(1.252-2.206) |
|  | rs9577924 | TT | 266 | (0.646) | 345 | (0.565) | 0.01 | NS | 1.405(1.086-1.817) |
|  |  | TC | 128 | (0.311) | 238 | (0.39) | 0.01 | NS | 0.706(0.542-0.920) |
|  |  | CC | 18 | (0.044) | 28 | (0.046) | 0.871 | NS | 0.951(0.519-1.743) |
|  |  | T | 660 | (0.801) | 928 | (0.759) | 0.027 | NS | 1.275(1.028-1.582) |
|  | rs9604466 | AA | 278 | (0.675) | 412 | (0.674) | 0.988 | NS | 1.002(0.768-1.308) |
|  |  | AG | 122 | (0.296) | 174 | (0.285) | 0.695 | NS | 1.057(0.803-1.391) |
|  |  | GG | 12 | (0.029) | 25 | (0.041) | 0.322 | NS | 0.703(0.349-1.416) |
|  |  | A | 678 | (0.823) | 998 | (0.817) | 0.724 | NS | 1.042(0.828-1.312) |
|  | rs9604488 | GG | 122 | (0.296) | 159 | (0.26) | 0.202 | NS | 1.199(0.908-1.583) |
|  |  | GA | 213 | (0.517) | 317 | (0.518) | 0.975 | NS | 0.996(0.776-1.279) |
|  |  | AA | 77 | (0.187) | 136 | (0.222) | 0.172 | NS | 0.804(0.589-1.100) |
|  |  | G | 457 | (0.555) | 635 | (0.519) | 0.111 | NS | 1.155(0.967-1.379) |
| PROS1 | rs12634349 | GG | 140 | (0.34) | 206 | (0.337) | 0.915 | NS | 1.014(0.779-1.321) |
|  |  | GA | 204 | (0.495) | 297 | (0.485) | 0.757 | NS | 1.040(0.810-1.335) |
|  |  | AA | 68 | (0.165) | 109 | (0.178) | 0.588 | NS | 0.912(0.654-1.272) |
|  |  | G | 484 | (0.587) | 709 | (0.579) | 0.714 | NS | 1.034(0.864-1.237) |
|  | rs13062355 | AA | 168 | (0.409) | 241 | (0.394) | 0.632 | NS | 1.064(0.825-1.373) |
|  |  | AG | 193 | (0.47) | 298 | (0.487) | 0.586 | NS | 0.933(0.726-1.198) |
|  |  | GG | 50 | (0.122) | 73 | (0.119) | 0.909 | NS | 1.023(0.697-1.501) |
|  |  | A | 529 | (0.644) | 780 | (0.637) | 0.771 | NS | 1.028(0.855-1.236) |
|  | rs4857037 | AA | 363 | (0.881) | 476 | (0.778) | 2.52×10^-5^ | 2.37×10^-3^ | 2.117(1.486-3.016) |
|  |  | AG | 48 | (0.117) | 131 | (0.214) | 5.57×10^-5^ | 5.24×10^-3^ | 0.484(0.339-0.692) |
|  |  | GG | 1 | (0.002) | 5 | (0.008) | 0.238 | NS | 0.295(0.034-2.537) |
|  |  | A | 774 | (0.939) | 1083 | (0.885) | 3.18×10^-5^ | 2.99×10^-3^ | 2.015(1.441-2.819) |
|  | rs6803590 | AA | 235 | (0.57) | 370 | (0.605) | 0.275 | NS | 0.868(0.674-1.119) |
|  |  | AG | 150 | (0.364) | 205 | (0.335) | 0.337 | NS | 1.137(0.875-1.477) |
|  |  | GG | 27 | (0.066) | 37 | (0.06) | 0.742 | NS | 1.090(0.653-1.820) |
|  |  | A | 620 | (0.752) | 945 | (0.772) | 0.305 | NS | 0.897(0.729-1.104) |
|  | rs7616142 | TT | 373 | (0.905) | 552 | (0.902) | 0.858 | NS | 1.040(0.680-1.589) |
|  |  | TC | 39 | (0.095) | 60 | (0.098) | 0.858 | NS | 0.962(0.630-1.470) |
|  |  | CC | 0 | (0) | 0 | (0) | / | / |  |
|  |  | T | 785 | (0.953) | 1164 | (0.951) | 0.861 | NS | 1.038(0.686-1.568) |
|  | rs8178607 | CC | 340 | (0.825) | 487 | (0.802) | 0.358 | NS | 1.164(0.842-1.608) |
|  |  | CT | 68 | (0.165) | 109 | (0.18) | 0.548 | NS | 0.903(0.648-1.259) |
|  |  | TT | 4 | (0.01) | 11 | (0.018) | 0.274 | NS | 0.531(0.168-1.680) |
|  |  | C | 748 | (0.908) | 1083 | (0.892) | 0.25 | NS | 1.191(0.884-1.603) |

P_c_ value: the Bonferroni corrected P value; NS:not significant

**Table S3** Polymorphisms of GAS/rs9577873 and PROS1/rs4857037 with main clinical features in BD

| SNP | Clinical features | Genotype/ | BD with | (freq.) | BD without | (freq.) | P value | P_c_ value | OR(95%CI) |
| --- | --- | --- | --- | --- | --- | --- | --- | --- | --- |
|  |  | Allele |  |  |  |  |  |  |  |
| rs9577873 | Genital ulcer |  | n=494 |  | n=413 |  |  |  |  |
|  |  | CC | 417 | (0.844) | 331 | (0.801) | 0.092 | NS | 1.342(0.952-1.890) |
|  |  | CT | 72 | (0.146) | 77 | (0.186) | 0.1 | NS | 0.745(0.524-1.058) |
|  |  | TT | 5 | (0.01) | 5 | (0.012) | 0.776 | NS | 0.834(0.240-2.902) |
|  |  | C | 906 | (0.917) | 739 | (0.895) | 0.103 | NS | 1.301(0.947-1.786) |
|  | Arthritis |  | n=135 |  | n=772 |  |  |  |  |
|  |  | CC | 110 | (0.815) | 638 | (0.826) | 0.743 | NS | 0.924(0.576-1.482) |
|  |  | CT | 24 | (0.178) | 125 | (0.162) | 0.646 | NS | 1.119(0.692-1.810) |
|  |  | TT | 1 | (0.007) | 9 | (0.012) | 0.663 | NS | 0.633(0.080-5.035) |
|  |  | C | 244 | (0.904) | 1401 | (0.907) | 0.848 | NS | 0.958(0.617-1.486) |
|  | Hypopyon |  | n=233 |  | n=674 |  |  |  |  |
|  |  | CC | 188 | (0.807) | 560 | (0.831) | 0.406 | NS | 0.850(0.580-1.247) |
|  |  | CT | 42 | (0.18) | 107 | (0.159) | 0.445 | NS | 1.165(0.787-1.726) |
|  |  | TT | 3 | (0.013) | 7 | (0.01) | 0.754 | NS | 1.243(0.319-4.846) |
|  |  | C | 418 | (0.897) | 1227 | (0.91) | 0.397 | NS | 0.859(0.604-1.221) |
|  | Skin lesions |  | n=648 |  | n=259 |  |  |  |  |
|  |  | CC | 547 | (0.844) | 201 | (0.776) | 0.015 | NS | 1.563(1.089-2.242) |
|  |  | CT | 92 | (0.142) | 57 | (0.22) | 0.004 | NS | 0.586(0.406-0.847) |
|  |  | TT | 9 | (0.014) | 1 | (0.004) | 0.191 | NS | 3.634(0.458-28.827) |
|  |  | C | 1186 | (0.915) | 459 | (0.886) | 0.055 | NS | 1.386(0.992-1.936) |
|  | Positive pathergy test |  | n=192 |  | n=715 |  |  |  |  |
|  |  | CC | 155 | (0.807) | 593 | (0.829) | 0.475 | NS | 0.862(0.573-1.296) |
|  |  | CT | 34 | (0.177) | 115 | (0.161) | 0.59 | NS | 1.123(0.737-1.710) |
|  |  | TT | 3 | (0.016) | 7 | (0.01) | 0.492 | NS | 1.605(0.411-6.268) |
|  |  | C | 344 | (0.896) | 1301 | (0.91) | 0.403 | NS | 0.853(0.587-1.240) |
| rs4857037 | Genital ulcer |  | n=494 |  | n=413 |  |  |  |  |
|  |  | AA | 428 | (0.866) | 364 | (0.881) | 0.5 | NS | 0.873(0.588-1.296) |
|  |  | AG | 63 | (0.128) | 47 | (0.114) | 0.528 | NS | 1.138(0.761-1.703) |
|  |  | GG | 3 | (0.006) | 2 | (0.005) | 0.803 | NS | 1.256(0.209-7.550) |
|  |  | A | 919 | (0.93) | 775 | (0.938) | 0.49 | NS | 0.876(0.603-1.274) |
|  | Arthritis |  | n=135 |  | n=772 |  |  |  |  |
|  |  | AA | 112 | (0.83) | 680 | (0.881) | 0.099 | NS | 0.659(0.400-1.085) |
|  |  | AG | 22 | (0.163) | 88 | (0.114) | 0.108 | NS | 1.513(0.911-2.515) |
|  |  | GG | 1 | (0.007) | 4 | (0.005) | 0.747 | NS | 1.433(0.159-12.918) |
|  |  | A | 246 | (0.911) | 1448 | (0.938) | 0.103 | NS | 0.680(0.426-1.084) |
|  | Hypopyon |  | n=233 |  | n=674 |  |  |  |  |
|  |  | AA | 201 | (0.863) | 591 | (0.877) | 0.575 | NS | 0.882(0.569-1.367) |
|  |  | AG | 31 | (0.133) | 79 | (0.117) | 0.523 | NS | 1.156(0.741-1.804) |
|  |  | GG | 1 | (0.004) | 4 | (0.006) | 0.77 | NS | 0.722(0.080-6.492) |
|  |  | A | 433 | (0.929) | 1261 | (0.935) | 0.638 | NS | 0.905(0.598-1.371) |
|  | Skin lesions |  | n=648 |  | n=259 |  |  |  |  |
|  |  | AA | 559 | (0.863) | 233 | (0.9) | 0.131 | NS | 0.701(0.441-1.113) |
|  |  | AG | 85 | (0.131) | 25 | (0.097) | 0.149 | NS | 1.413(0.882-2.264) |
|  |  | GG | 4 | (0.006) | 1 | (0.004) | 0.671 | NS | 1.602(0.178-14.406) |
|  |  | A | 1203 | (0.928) | 491 | (0.948) | 0.129 | NS | 0.711(0.458-1.106) |
|  | Positive pathergy test |  | n=192 |  | n=715 |  |  |  |  |
|  |  | AA | 164 | (0.854) | 628 | (0.878) | 0.372 | NS | 0.811(0.513-1.284) |
|  |  | AG | 26 | (0.135) | 84 | (0.117) | 0.499 | NS | 1.177(0.734-1.886) |
|  |  | GG | 2 | (0.01) | 3 | (0.004) | 0.307 | NS | 2.472(0.410-14.901) |
|  |  | A | 354 | (0.922) | 1340 | (0.937) | 0.288 | NS | 0.793(0.516-1.218) |

P_c_ value: the Bonferroni corrected P value; NS:not significant

**Table S4** Pairwise Linkage Disequilibrium tests (r^2^ value)of six SNPs in PROS1

| r^2^ | rs4857037 | rs7616142 | rs6803590 | rs8178607 | rs13062355 |
| --- | --- | --- | --- | --- | --- |
| rs12634349 | 0.245 | 0.079 | 0.506 | 0.1 | 0.707 |
| rs4857037 | - | 0.012 | 0.488 | 0.013 | 0.375 |
| rs7616142 | - | - | 0.039 | 0.009 | 0.065 |
| rs6803590 | - | - | - | 0.016 | 0.602 |
| rs8178607 | - | - | - | - | 0.14 |

**Table S5** Pairwise Linkage Disequilibrium tests (D' value) of six SNPs in PROS1

| D' | rs4857037 | rs7616142 | rs6803590 | rs8178607 | rs13062355 |
| --- | --- | --- | --- | --- | --- |
| rs12634349 | 0.999 | 0.998 | 0.914 | 0.999 | 0.936 |
| rs4857037 | - | 0.969 | 0.999 | 1 | 0.999 |
| rs7616142 | - | - | 0.997 | 0.911 | 0.999 |
| rs6803590 | - | - | - | 0.639 | 0.999 |
| rs8178607 | - | - | - | - | 0.916 |

**Table S6 Haplotype analysis of six SNPs in PROS1 between BD and control group**

| Haplotype | BD,n(freq) | Control,n(freq) | Chi2 | p value | OR(95%CI) |
| --- | --- | --- | --- | --- | --- |
| A A T A C A | 117.90(0.143) | 99.79(0.082) | 12.136 | 0.000499 | 1.660(1.246-2.212) |
| A A T A C G | 67.31(0.082) | 119.19(0.098) | 4.436 | 0.035239 | 0.712(0.519-0.978) |
| A A T G C G | 90.46(0.110) | 77.44(0.064) | 8.574 | 0.003425 | 1.610(1.168-2.219) |
| G A T A C A | 327.63(0.399) | 515.95(0.425) | 14.187 | 0.000167 | 0.684(0.561-0.834) |
| G A T G C A | 26.48(0.032) | 35.79(0.029) | 0.015 | 0.903591 | 0.969(0.580-1.617) |
| G A T A C G | 68.99(0.084) | 67.68(0.056) | 3.153 | 0.075861 | 1.373(0.967-1.950) |

Notes:The frequency of haplotype below 0.03 in both control & case was not included and the risk assessment was not performed;

The global haplotype frequencies were significantly different between case and control group (P<0.001). Haplotype of these SNPs

in the following order (left to right):rs12634349, rs4857037, rs7616142, rs6803590, rs8178607, rs13062355.

**Table S7** Pairwise Linkage Disequilibrium tests (r^2^ value) of twelve SNPs in GAS6

| r^2^ | rs7994900 | rs7492052 | rs6602910 | rs12868833 | rs7319547 | rs7399860 | rs9577924 | rs7323932 | rs9604466 | rs9577873 | rs7399637 |
| --- | --- | --- | --- | --- | --- | --- | --- | --- | --- | --- | --- |
| rs9604488 | 0.37 | 0.165 | 0.682 | 0.014 | 0.119 | 0.04 | 0.251 | 0.076 | 0.1 | 0.068 | 0.145 |
| rs7994900 | - | 0.09 | 0.537 | 0.072 | 0.065 | 0.011 | 0.559 | 0.004 | 0.234 | 0.036 | 0.058 |
| rs7492052 | - | - | 0.069 | 0.008 | 0.679 | 0.022 | 0.049 | 0.199 | 0.012 | 0.428 | 0.059 |
| rs6602910 | - | - | - | 0.031 | 0.133 | 0.067 | 0.33 | 0.079 | 0.126 | 0.1 | 0.214 |
| rs12868833 | - | - | - | - | 0.009 | 0.009 | 0.133 | 0.007 | 0.102 | 0.01 | 0.025 |
| rs7319547 | - | - | - | - | - | 0.043 | 0.034 | 0.262 | 0.008 | 0.539 | 0.09 |
| rs7399860 | - | - | - | - | - | - | 0.154 | 0.036 | 0.076 | 0.063 | 0.179 |
| rs9577924 | - | - | - | - | - | - | - | 0.005 | 0.408 | 0.031 | 0.109 |
| rs7323932 | - | - | - | - | - | - | - | - | 0.012 | 0.161 | 0.023 |
| rs9604466 | - | - | - | - | - | - | - | - | - | 0.017 | 0.362 |
| rs9577873 | - | - | - | - | - | - | - | - | - | - | 0.251 |

**Table S8** Pairwise Linkage Disequilibrium tests (D' value) of twelve SNPs in GAS6

| D' | rs7994900 | rs7492052 | rs6602910 | rs12868833 | rs7319547 | rs7399860 | rs9577924 | rs7323932 | rs9604466 | rs9577873 | rs7399637 |
| --- | --- | --- | --- | --- | --- | --- | --- | --- | --- | --- | --- |
| rs9604488 | 0.85 | 0.877 | 0.877 | 0.365 | 0.845 | 0.291 | 0.878 | 0.47 | 0.657 | 0.659 | 0.526 |
| rs7994900 | - | 0.882 | 0.976 | 0.759 | 0.846 | 0.241 | 0.955 | 0.14 | 0.733 | 0.647 | 0.271 |
| rs7492052 | - | - | 0.548 | 0.282 | 0.952 | 0.427 | 0.812 | 0.599 | 0.434 | 0.783 | 0.407 |
| rs6602910 | - | - | - | 0.618 | 0.858 | 0.384 | 0.963 | 0.459 | 0.705 | 0.766 | 0.608 |
| rs12868833 | - | - | - | - | 0.279 | 0.285 | 0.833 | 0.258 | 0.621 | 0.278 | 0.426 |
| rs7319547 | - | - | - | - | - | 0.681 | 0.757 | 0.775 | 0.343 | 0.777 | 0.56 |
| rs7399860 | - | - | - | - | - | - | 0.925 | 0.433 | 0.764 | 0.841 | 0.775 |
| rs9577924 | - | - | - | - | - | - | - | 0.181 | 0.769 | 0.737 | 0.447 |
| rs7323932 | - | - | - | - | - | - | - | - | 0.353 | 0.629 | 0.206 |
| rs9604466 | - | - | - | - | - | - | - | - | - | 0.616 | 0.934 |
| rs9577873 | - | - | - | - | - | - | - | - | - | - | 0.952 |
